# Postnatal Steroids in Preterm Infants: A Narrative Review Series—Part 2: Cardiovascular Impacts

**DOI:** 10.3390/children13030395

**Published:** 2026-03-12

**Authors:** Phoenix Plessas-Azurduy, Anie Lapointe, Punnanee Wutthigate, Sarah Spénard, Andréanne Villeneuve, Audrey Hébert, Eilon Shany, Justin Richardson, Neta Geva, Wadi Mawad, Tiscar Cavallé-Garrido, Marc Beltempo, Wissam Shalish, Guilherme Sant’Anna, Gabriel Altit

**Affiliations:** 1 Division of Clinical & Translational Research, Faculty of Medicine and Health Sciences, McGill University, Montreal, QC H3A 0G4, Canada; phoenix.plessas-azurduy@mail.mcgill.ca; 2 Division of Neonatology, Department of Pediatrics, Université de Montréal, CHU Sainte-Justine, Montréal, QC H3T 1J4, Canada; anie.lapointe@umontreal.ca (A.L.); andreanne.villeneuve@umontreal.ca (A.V.); 3 Department of Pediatrics, Faculty of Medicine Siriraj Hospital, Mahidol University, Bangkok 10700, Thailand; punnanee.wut@mahidol.edu; 4 Division of Neonatology, Department of Pediatrics, Montreal Children’s Hospital, Montreal, QC H4A 3H9, Canada; sarah.spenard.med@ssss.gouv.qc.ca (S.S.); marc.beltempo@mcgill.ca (M.B.); wissam.shalish@mcgill.ca (W.S.); guilherme.santanna@mcgill.ca (G.S.); 5 Division of Neonatology, Department of Pediatrics, CHU de Québec, Laval University, Québec, QC G1V 0A6, Canada; audrey.hebert.2@ulaval.ca; 6 Department of Neonatology, Saban Children’s Hospital, Soroka Medical Center, Beer Sheva 84101, Israel; eshany@bgu.ac.il (E.S.); richardsonj@doctors.net.uk (J.R.); gevan@post.bgu.ac.il (N.G.); 7 Faculty of Health Sciences, Ben Gurion University of the Negev, Beer Sheva 84105, Israel; 8 Division of Cardiology, Department of Pediatrics, Montreal Children’s Hospital, Montreal, QC H4A 3H9, Canada; wadi.mawad@mcgill.ca (W.M.); tiscar.cavalle2@muhc.mcgill.ca (T.C.-G.)

**Keywords:** corticosteroids, extremely preterm infants, cardiovascular physiology, patent ductus arteriosus, myocardial remodeling, autonomic regulation, pulmonary hypertension, targeted neonatal echocardiography, heart rate variability, individualized steroid therapy

## Abstract

**Highlights:**

**What are the main findings?**

- Systemic postnatal corticosteroid use in extremely preterm infants with evolving lung disease can impact cardiovascular structure and function. Current evidence suggests it can lead to reactive myocardial hypertrophy, potentially accelerated closure of the patent ductus arteriosus and alterations in autonomic regulation and vascular resistance.
- Emerging diagnostic tools provide real-time monitoring capabilities: advanced techniques such as speckle tracking echocardiography, heart rate variability analysis, and biofluid markers (e.g., BNP) can detect subclinical cardiovascular changes and steroid-induced stress.

**What are the implications of the main findings?**

- Routine cardiovascular surveillance is essential during steroid therapy to identify and manage potential adverse effects, such as left ventricular outflow tract obstruction or systemic hypertension, which may complicate the clinical course of infants with evolving bronchopulmonary dysplasia.
- Physiological data should guide a shift toward precision-based medicine, where the integration of longitudinal imaging and biomarker monitoring enables individualized steroid timing and dosing to optimize therapeutic benefits while minimizing unintended cardiovascular harm.

**Abstract:**

Postnatal corticosteroids are frequently administered to extremely preterm infants to support respiratory management, yet their effects on the immature cardiovascular system are complex and underexplored. As the second installment in a series on physiology-informed steroid use, this narrative review focuses on the cardiovascular consequences of systemic corticosteroid therapy in preterm neonates. We examine how corticosteroids influence key aspects of cardiovascular physiology, including ductal closure, systemic and pulmonary vascular resistance, myocardial remodeling, and autonomic regulation. Attention is given to the hemodynamic transition of early postnatal life and how steroid exposure may interact with patency of the ductus arteriosus and vascular development. The potential for corticosteroids to contribute to reactive myocardial hypertrophy, systemic hypertension, and pulmonary hypertension is also reviewed in the context of both short- and long-term outcomes. Emerging diagnostic and monitoring tools are discussed for their potential to guide individualized therapy. These include targeted neonatal echocardiography (TnECHO) to assess cardiac function and structure, electrocardiography (ECG) for rhythm and conduction abnormalities, heart rate variability analysis for autonomic function, and circulating biomarkers to evaluate myocardial stress and inflammation. Together, these tools may inform tailored steroid timing and dosing, especially in the research context, while monitoring for signs of cardiovascular side effects in real time. By synthesizing mechanistic insights with evolving clinical evidence, this review highlights the need for a more nuanced understanding of how corticosteroids affect the developing cardiovascular system. It underscores the importance of integrating cardiovascular monitoring into routine care to optimize therapeutic benefit while minimizing unintended harm. Alongside companion reviews addressing respiratory and growth impacts, this installment contributes to a broader framework for individualized, physiology-driven steroid use in extremely preterm infants.

## 1. Introduction

The role of systemic postnatal corticosteroids in the care of extremely preterm infants remains highly controversial [1,2,3]. Although these medications are commonly administered beyond the first postnatal week to promote extubation or weaning of invasive ventilation and manage evolving bronchopulmonary dysplasia (BPD), their effects on cardiovascular physiology are not as clearly defined [4,5,6]. Clinical practices surrounding the initiation, dosing, and monitoring of postnatal corticosteroids (PNS) vary widely across neonatal centers, reflecting the lack of standardized guidelines [7,8,9]. Research has drawn attention to the impact of systemic PNS on cardiovascular structure and function such as effects on the myocardium [10], associations with ductal constriction [11] and reactive hypertrophic cardiomyopathy [1], but causality remains uncertain. Additionally, high-dose postnatal steroid use in animal studies have been associated with substantial disruptions in cardiac development, including altered gene expression, impaired cardiomyocyte maturation, reduced right-ventricular cell numbers, and increased left-ventricular inflammation, whereas low-dose exposure produced minimal effects [10]. At the same time, innovations in neonatal cardiovascular imaging and physiologic assessment have created new opportunities to better understand the systemic effects of these agents. This review synthesizes current literature on the cardiovascular consequences of systemic PNS use in preterm infants and highlights emerging and available tools for monitoring these effects that may enable research avenues for more individualized and developmentally informed approaches to their use in preterm infants.

## 2. Documented Effects of Postnatal Steroids on the Cardiovascular System

### 2.1. Reactive Hypertrophic Cardiomyopathy

Exogenous administration of glucocorticoids, including dexamethasone and hydrocortisone, have been associated with altered cardiac structure, particularly reactive ventricular hypertrophy by increased cardiomyocyte protein synthesis leading to increased cell size via glucocorticoid receptors [12,13,14,15,16,17,18]. This structural change seems transient [12,19,20,21] but can theoretically impact cardiac performance, primarily by restricting relaxation properties. Additionally, reactive hypertrophy may cause dynamic left ventricular outflow tract (LVOT) obstruction during contraction [22,23,24]. Other potential effects include sub-aortic septal thickening [25] and systolic anterior motion of the mitral valve [19], both of which can contribute to LVOT obstruction [19]. Impaired ventricular relaxation can lead to elevated left ventricular (LV) filling pressure, which in turn increases left atrial pressure and pulmonary venous pressure [26]. Unless a large interatrial shunt is present to decompress the left atrium experiencing rising pressure, this may disrupt pulmonary venous drainage and mechanics [27]. The resulting elevated pulmonary venous pressure may contribute to increased alveolar fluid leakage (“spillage”), potential pulmonary edema [28]. However, this potential side effect should be weighed against the side effects of not treating with postnatal steroids, where inflammatory reactions at the alveolar level, as well as heterogenous collapse due to reduced pulmonary compliance, may also contribute to pulmonary densification [29].

**Figure 1:**
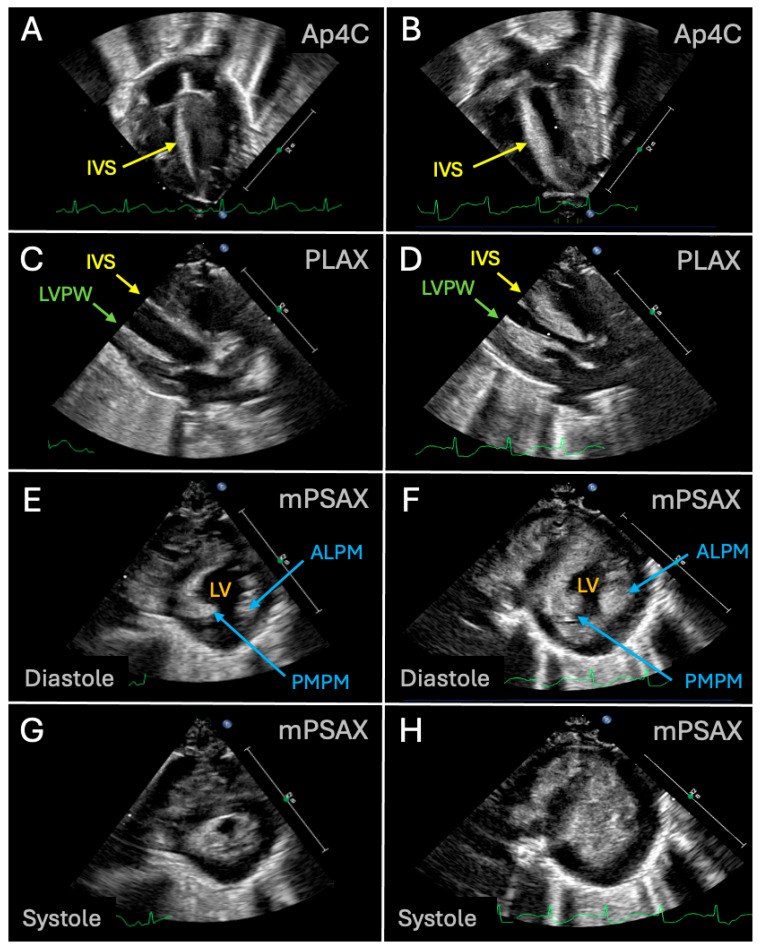
Conventional 2D echocardiographic images of a neonatal heart (left: (**A**,**C**,**E**,**G**)) and one with reactive hypertrophic cardiomyopathy (right: (**B**,**D**,**F**,**H**)). Of note: (**D**) LV obstruction due to concomitant reactive hypertrophy of the IVS and LVPW and (**F**,**H**) uniform, concentric reactive LV hypertrophy as shown on the mPSAX view. Abbreviations: Ap4C (apical four-chamber view); IVS (interventricular septum); PLAX (parasternal long-axis view); LVPW (left ventricular posterior wall); ALPM (anterolateral papillary muscle); PMPM (posteromedial papillary muscle); mPSAX (modified parasternal short-axis view).

Reactive hypertrophic cardiomyopathy and hypertrophy of the interventricular septum (IVS) (Figure 1) have frequently been reported in preterm infants treated with a high cumulative dose of dexamethasone (i.e.,: 5 to 8 mg/kg) [19,30,31,32,33].

Echocardiography remains the primary modality for evaluating cardiac structural and functional changes in preterm infants exposed to systemic postnatal corticosteroids [34]. Motion (M)-mode imaging is used for measurement of ventricular wall thickness and chamber dimensions [35], allowing for the identification of reactive hypertrophic changes such as interventricular septal thickening and left ventricular posterior wall hypertrophy [34]. Doppler studies complement this by assessing flow dynamics, enabling the detection of turbulent flow and pressure gradients suggestive of LVOT obstruction [36]. Together, these modalities provide essential, noninvasive insight into the cardiovascular effects of corticosteroids during a period of rapid myocardial remodeling. Previous echocardiography studies have reported a significant increase in septal and LV posterior wall (LVPW) thickness (by M-mode) and a decrease in LV end diastolic diameter (LVEDD—by M-mode) in newborns exposed to postnatal steroids [30,31,32]. In one study, two infants developed Doppler evidence of LVOT obstruction, without clinical signs of decreased systemic perfusion [32]. Zecca et al. also described the cardiac adverse effects of early dexamethasone treatment in preterm infants born at ≤1250 g and ≤30 weeks GA [25], when compared to controls. In their study, dexamethasone was initiated at day of life 4 at 0.5 mg/kg, which was tapered over the following 7 days, for a total cumulative dose of 2.375 mg/kg. Echocardiography was done longitudinally: (a) before treatment initiation, (b) at 3- to 7-days after initiation, (c) at 7 days after dexamethasone discontinuation, and (d) at 28 days of life. M-mode echocardiography markers of reactive LV hypertrophy were present, such as increased thickness of the IVS and LV posterior wall, both at end-diastole and end-systole, at 7 days after initiation and 7 days after discontinuation. Four neonates (20%) treated with dexamethasone developed reactive LV myocardial hypertrophy without frank LVOT obstruction, but Doppler revealed mild flow acceleration in the LVOT, with a mean Vmax of 1.87 m/s (range: 1.73–1.92 m/s). The reactive LV hypertrophy resolved within two to three weeks after steroids discontinuation.

In another study, Skelton et al. investigated 31 premature infants (23 to 34 weeks GA at birth) receiving 0.4–0.6 mg/kg/day cumulative dose of dexamethasone for chronic lung disease initiated at a median age of 11 days (range 2–34 days) and weaned over 2 to 3 weeks [37]. A total of 29 (94%) infants developed concentric (Figure 1), uniform reactive LV hypertrophy beginning on the third day of treatment. The average increase in ventricular wall thickness by using M-mode was between 55–67% but 3 patients had an increase greater than 100%. There was no significant LVOT obstruction. Only 15 (48%) patients were available for follow-up and all of them showed complete resolution of the reactive LV hypertrophy by day 27.

From the available data, reactive myocardial hypertrophy does not seem to be exclusively related to the cumulative dose or duration of dexamethasone administration. In one case report, a significant increase in thickness of the IVS was reported after exposure to a single dose of dexamethasone (0.5 mg/kg) [33]. Furthermore, in the “Minidex” study, reactive IVS hypertrophy was noted in 24- and 25-week infants even with a very low dose of dexamethasone of 0.05 mg/kg/day [38]. Additionally, in a case series [39], two extremely preterm infants exposed to very-low-dose dexamethasone developed clinical signs of low cardiac output with new-onset systolic ejection murmur and poor peripheral perfusion upon echocardiographic assessment. No serious adverse events were documented (no acidosis, inotrope use or death). One of these infants showed severe obstruction within the LV cavity, between the hypertrophied IVS and the basal aspect of the anterior papillary muscle, with a Doppler derived gradient of 110 mmHg. The other infant had reactive myocardial hypertrophy and interventricular stenosis—described by the authors as a dynamic stenosis between the basal hypertrophic left anterior papillary muscle and the intraventricular septum, in which blood flow acceleration originates. Both infants were treated with beta-blocker therapy to promote ventricular relaxation [39].

**Figure 2:**
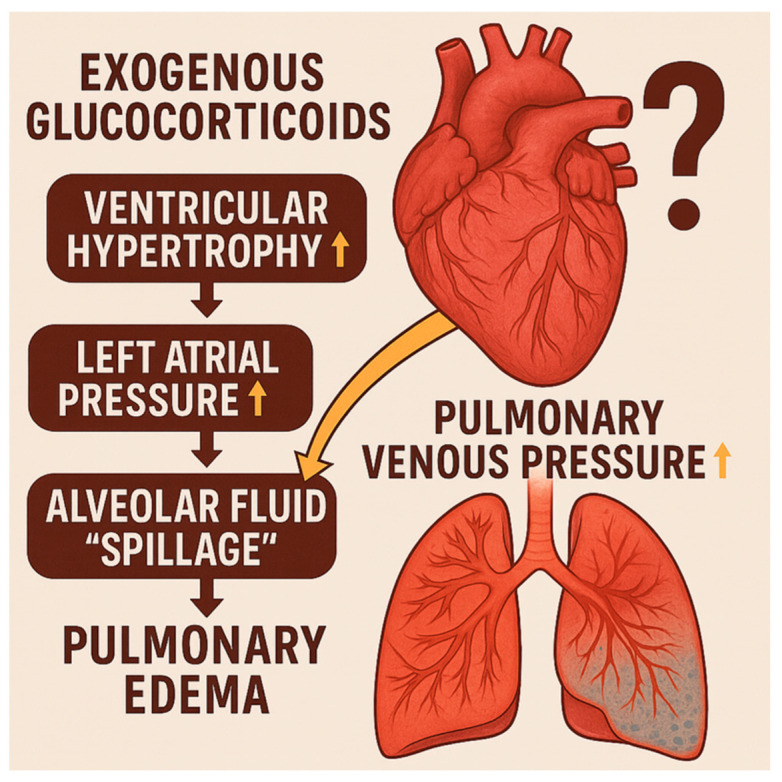
Proposed mechanistic link between exogenous glucocorticoids, reactive ventricular hypertrophy and pulmonary edema in preterm infants with evolving BPD. Up arrow indicates an increase.

In addition, preterm infants who develop reactive LV hypertrophy in response to postnatal corticosteroid use may be at risk of increased LV end diastolic pressure and increased LA pressure, which, in turn, may contribute to increased pulmonary venous pressure and pulmonary edema, potentially worsening pulmonary compliance (Figure 2) [40]. This could have important downstream effects on both short- and long-term cardiorespiratory outcomes, including prolonged ventilator dependence, impaired oxygenation, and potential remodeling of the pulmonary vasculature, as outlined above. As such, some infants may be experiencing these concerning responses, and future studies should aim to identify which patients are affected and determine whether the occurrence of reactive hypertrophy warrants adjusting or even discontinuing steroid treatment.

While the reactive hypertrophic effects of systemic postnatal corticosteroids on cardiac structure are becoming increasingly recognized, their influence on functional cardiovascular physiology extends beyond the myocardium. Notably, these agents have also been implicated in modulating ductal closure dynamics [11], raising important questions about how they interact with persistent patency of the ductus arteriosus in the context of evolving lung disease.

### 2.2. Persistence of the Patent Ductus Arteriosus

Extremely premature newborns are at high risk of persistent ductus arteriosus (PDA), a fetal connection between the aorta and pulmonary artery that maintains patency in many preterm infants due to immature closure mechanisms [41]. The PDA may expose the pulmonary vasculature to systemic pressure and to excessive flow potentially contributing to pulmonary edema, by the increase in pulmonary blood flow overwhelming the immature pulmonary vasculature (in the context of reduced LV compliance raising LA pressure) [42]. Dexamethasone possibly contributes to the acceleration of PDA closure [43,44], and may also diminish pulmonary edema through this mechanism. The role of dexamethasone in PDA closure remains a topic of ongoing research, with evidence suggesting potential benefits but also highlighting gaps in understanding the underlying mechanisms and clinical implications. Sehgal et al. investigated the cardiovascular effects of a low-dose dexamethasone regimen (DART) in extremely preterm infants, specifically its impact on the PDA and pulmonary circulation [45]. Among the 30 infants studied, 20 had a PDA at baseline echocardiography. Authors found a temporal association between dexamethasone use and reduction in ductal diameter (from 2.16 ± 0.8 mm to 1.1 ± 0.8 mm, *p* = 0.0003) with complete closure in 35% of cases. Additionally, pulmonary vascular resistance decreased, as indicated by an increased time to peak velocity/right ventricular ejection time ratio, and right ventricular systolic function improved as assessed by echocardiography performed within 24 h of the completion of a 10-day DART course. These findings suggest that dexamethasone may contribute to PDA closure while also enhancing pulmonary hemodynamics, potentially reducing pulmonary edema by limiting excessive left-to-right shunting. However, a significant confounder of these findings is that this population was not managed with a conservative approach to PDA. Conservative/expectant management typically relies on watchful waiting, whereas non-conservative strategies often involve early use of non-steroidal anti-inflammatory drugs (NSAIDs) or acetaminophen use to pharmacologically accelerate ductal closure. As a result, some infants within this study population received NSAIDs, potentially confounding the observed results. Prior to this, in 1990, Heyman et al. investigated the effects of corticosteroids on PDA tone in preterm infants [46]. They reported 4 cases of premature infants in whom there was a temporal association between dexamethasone administration and ductal closure. Their findings indicated that steroids promote ductal constriction, which could be attributed to their modulation of prostaglandin metabolism (by interfering with prostaglandin synthesis or reducing sensitivity of the smooth muscle cells to prostaglandin), a key factor in maintaining ductal patency. In 1998, Morales et al. examined early dexamethasone therapy in preterm infants with a birth weight between 700 and 999 g with severe respiratory distress syndrome [47]. Infants received either a 12-day course of dexamethasone (*n* = 13) or placebo (n = 17) within 12 h of birth. PDA incidence was lower in the dexamethasone group (23% vs. 59%, *p* = 0.05) as well as number of days infants required ventilation, oxygen needs, and BPD incidence as compared to placebo. These findings suggest that dexamethasone may promote PDA closure, reducing pulmonary overload and BPD risk. Eronen et al. described that antenatal dexamethasone did not constrict the fetal ductus arteriosus but increased spontaneous postnatal closure in preterm infants born ≤30 weeks [48]. They evaluated fetal ductal flow by Doppler echocardiography which showed no differences in flow between groups, but postnatal closure was more frequent in the dexamethasone group. These findings suggest that while antenatal dexamethasone does not affect the fetal ductus, it may facilitate postnatal closure in very preterm infants. Tsai et al. reported that dexamethasone may promote PDA closure by upregulating 15-hydroxy-prostaglandin dehydrogenase (15-PGDH), the enzyme responsible for inactivating PGE2 [49]. Their findings suggest that dexamethasone may accelerate ductal constriction by reducing PGE2 activity, a key regulator of ductal patency. On the other hand, Smith et al. concluded that advancing GA and birth may inhibit relaxation of the ductus through a corticosteroid-independent reduction in EP4 receptor gene expression [50]. They investigated the effects of corticosteroids, GA, and birth on the expression of prostanoid receptor genes (EP3 and EP4) in the ductus arteriosus of lambs and baboons. EP4 receptors mediate prostaglandin E2 (PGE2)-induced relaxation of the ductus arteriosus, while EP3 receptors counteract this effect. The study found that EP4 expression decreased with advancing GA and after birth, reducing PGE2-mediated relaxation, whereas EP3 expression remained unchanged. Corticosteroids had no effect on either receptor, indicating that the decline in EP4 expression and its impact on PGE2 function are independent of corticosteroid exposure. Additionally, the PREMILOC study group investigated whether prophylactic low-dose hydrocortisone (1 mg/kg/day for 7 days, then 0.5 mg/kg/day for 3 days) improved survival without BPD in extremely preterm infants (<28 weeks’ GA). The hydrocortisone group (n = 255) had a significantly higher rate of survival without BPD at 36 weeks MA compared to placebo, with fewer cases of PDA requiring ligation, suggesting hydrocortisone, via its corticosteroid properties, may contribute to accelerating ductal closure. While dexamethasone and other corticosteroids show promise in promoting ductal constriction in preterm infants and, eventually, closure, as well as improving outcomes in preterm infants, the mechanisms remain incompletely understood, and further research is necessary to fully elucidate their effects and optimize clinical application.

The Trial of Indomethacin Prophylaxis in Preterms (TIPP) investigated prophylactic use of indomethacin compared to placebo and demonstrated that rates of BPD in both the indomethacin and placebo subgroups without a PDA were 43% (170/391) after indomethacin prophylaxis and 30% (78/257) after placebo (*p* [interaction] = 0.015) [51]. Researchers posited that indomethacin may not prevent BPD (although efficacious in reducing the PDA) due to harmful side effects on oxygenation and edema formation. In addition, a 2025 meta-analysis looking at active (pharmacologic or surgical, though primarily pharmacologic (via NSAIDs or Acetaminophen) versus expectant management of PDA found that active treatment during the first 2 weeks of life was associated with a significantly higher incidence of death or moderate–severe BPD, and increased mortality than the expectant management approach [52]. This may be mediated by side effects of the medications, pulmonary toxicity, multi-organ prolonged vasoconstriction and hypoperfusion, as well as non-efficacy regarding permanent PDA restriction and/or closure. Additionally, catheter closure for the PDA is currently undergoing testing for efficacy by RCT. As such, dexamethasone should be considered in infants with evolving lung disease as early as the second week of life and may simultaneously, or secondarily, help with ductal closure in those patients with concurrent (hemodynamically significant or large) PDAs.

Future trials should specifically address this question and outline whether this strategy confers an advantage in terms of measurable meaningful outcomes regarding pulmonary health, mortality and long-term neurodevelopment in these infants.

### 2.3. Hypertension and Vascular Stiffness

Rates of systemic hypertension in infants with BPD are much higher in the neonatal population, although why this prevalence exists remains unclear [53]. Hypertension linked to BPD was first identified by Abman in the mid-1980s, who observed that 43% of infants with BPD developed hypertension, compared to just 4.5% of those without BPD [54]. Notably, more than half of these cases were only recognized after NICU discharge, highlighting the importance of continued blood pressure surveillance in this high-risk group [55]. Subsequent research has consistently shown that hypertension is more prevalent among infants with BPD and that the likelihood increases with greater BPD severity with an almost 5-fold increase in the odds of infantile hypertension associated with BPD secondary to prematurity being described by multiple groups [56,57,58,59]. Although this association has been recognized for decades now, it is still uncertain whether BPD itself directly contributes to the development of hypertension through a specific biological mechanism, or if BPD mainly reflects the presence of other underlying risk factors that predispose infants to elevated blood pressure. In addition, infants with neonatal hypertension have been found to have a significantly higher incidence of BPD, use of PNS and patent ductus arteriosus than those without hypertension [60].

Postnatal corticosteroids are clinically utilized to acutely raise blood pressure in infants with refractory hypotension by increasing systemic vascular resistance and enhancing tissue perfusion [61]. While these effects can be transient, infants treated with postnatal steroids show a significantly higher incidence of neonatal hypertension, a condition often associated with the severity of BPD. However, longitudinal data in humans suggests that long-term blood pressure trajectories are not adversely affected, as extended tapering courses of dexamethasone did not result in significant differences in systolic blood pressure at school-age follow-up [62]. It should be noted that evidence regarding vascular stiffness in the context of postnatal steroids is largely extrapolated from animal models, and clinical data in preterm neonates remains limited.

Postnatal corticosteroids exert a range of systemic vascular effects in infants with evolving BPD, some of which may be transient while others persist longer-term. Emerging evidence, largely derived from animal models, suggests that glucocorticoids influence vascular stiffness and tone in preterm infants primarily through modulation of endothelial nitric oxide synthase (eNOS) and nitric oxide (NO) signaling pathways [63]. In animal studies, short courses of dexamethasone appear to protect against hypoxia-induced pulmonary hypertension by preserving eNOS mRNA expression and Akt phosphorylation, supporting eNOS activation and NO production in pulmonary arteries [64]. More broadly, corticosteroids can acutely increase systemic vascular resistance via non-transcriptional eNOS activation, enhancing NO generation and tissue perfusion, independent of eNOS gene expression—effects observed in adult models of stroke and myocardial infarction [65,66]. However, prolonged glucocorticoid exposure has been shown to downregulate eNOS transcription and reduce NO bioavailability in organs such as the liver and kidney, contributing to sustained elevations in vascular tone [67,68]. While these mechanisms are well-described in animal and adult models, clinical data regarding their direct impact on vascular stiffness in preterm neonates remains limited.

Antenatal glucocorticoids, including dexamethasone [69,70] and betamethasone [71,72], have been shown in animal models to increase eNOS expression and support pulmonary vascular adaptation at birth, though whether these mechanisms extend to the postnatal period remains uncertain [73]. Short-term, high-dose postnatal glucocorticoid treatment can enhance eNOS phosphorylation and cyclic GMP production, promoting vasodilation [74,75], but these effects appear to diminish with prolonged exposure [76]. In both neonatal and adult animal models, steroids have demonstrated protective effects against pulmonary hypertension, possibly by maintaining eNOS activity and limiting oxidative stress [64]. Despite these findings, their relevance to human preterm infants remains unclear, with limited data on the duration or clinical impact of such vascular tone changes. Experimental animal studies suggest that glucocorticoids can enhance regional blood flow in the kidney, heart, and eye [74,75]. For example, a week-long dexamethasone regimen in adult rats led to renal vasodilation and increased eNOS expression [76].

At the kidney level, however, there is evidence that postnatal steroids may disrupt nephron development: in neonatal rats, early dexamethasone exposure decreased glomerular number and long-term renal function by promoting apoptosis [77]. Postnatal steroids can increase retinal blood flow in preterm infants [78] and have been linked to higher rates of adult systolic dysfunction in animal models [12]. Given the current reliance on data extrapolated from animal and adult models, further research is needed to clarify how dosing, timing, and duration of glucocorticoid exposure shape vascular outcomes and whether these effects contribute meaningfully to the benefits and risks of steroid use in this population.

### 2.4. Impacts on Heart Rate Variability

Corticosteroid exposure may influence autonomic nervous system (ANS) function [79]. Heart rate variability (HRV), a marker of ANS maturity and balance, reflects the heart’s ability to respond to autonomic signals [80,81,82].

Prematurity delays HRV maturation [83], with consistently lower time domain HRV metrics observed in preterm compared to term infants, indicating impaired autonomic regulation [84]. These effects may contribute to long-term developmental issues. HRV and heart rate characteristics (HRC) indices, which indicate risk of sepsis or decompensation, may help tailor oxygen strategies and monitor illness severity [82,85,86,87]. A higher HRC index reflects reduced HRV and abnormal patterns [88,89].

HRV is typically captured through short- or long-term data collection using electrocardiogram (ECG) recordings, which allow for detailed analysis of beat-to-beat variability and autonomic modulation.

The utility of HRV is well-established in fetal and adult medicine, providing a baseline for its emerging role in the NICU. During hypoxia, fetal HR regulation via the ANS compensates for limited stroke volume [90], and HRV changes may precede overt fetal distress [91]. Fetal HRV is an established marker of well-being [92,93], and in adult populations studies show HRV predicts outcomes across several conditions. In adult populations, glucocorticoid therapy has been shown to normalize HRV [94]. In neonates with BPD, high-frequency parasympathetic activity is significantly reduced—indicating chronic autonomic dysregulation [95]. Low HRV and high HRC index also appear in other conditions like respiratory failure [96,97,98]. Despite consistent findings, limited cohort sizes and heterogeneous protocols hinder clinical standardization.

Antenatal corticosteroids have been associated with transiently increased fetal HRV and reduced heart rate [80,99,100], with values returning to baseline after exposure ends. Mechanisms may include anti-inflammatory pathways. In a mouse model, dexamethasone increased HRV independent of inflammation, suggesting improved ANS function [101]. Experimental animal studies further show HRV sensitivity to hypoxia and inflammation, with emerging evidence that combining glucocorticoids with statins can normalize sympathetic signaling via NO without diminishing anti-inflammatory effects [102,103,104,105,106,107].

In the preterm population specifically, infants with BPD exhibit high-frequency parasympathetic reduction, indicating chronic autonomic dysregulation. In preterm neonates, HRV predicts prognosis and sepsis risk [82,85,86,87]. Lower HRV reflects impaired vagal tone and stress vulnerability, while higher HRV suggests better autonomic control [108]. Kaczmarek et al. found that infants failing extubation had significantly lower HRV, and dexamethasone significantly increased HRV in others [109]. However, HRV prior to extubation was not predictive of reintubation within 3 days [109].

Antenatal steroid use (beta- and dexamethasone) has also been associated with transient HRV suppression [100,110,111].

The HeRO monitor, developed by Moorman et al., uses HRV-derived HRC indices to detect sepsis within 24 h in preterm and VLBW infants [86,102]. However, data describing the specific effects of postnatal corticosteroids on HRV remain sparse and current clinical evidence consists primarily of small, observational cohorts. This is important given the known relationship between stress exposure, HPA axis activity, elevated cortisol levels, and inflammation in preterm infants [112,113,114], all of which impair ANS development and HRV [115].

Preliminary clinical data from one study in preterm infants with BPD on mechanical ventilation reported that dexamethasone was associated with increased HRV and reduced HRC index, likely through anti-inflammatory effects and potential upregulation of adrenergic and cholinergic signaling [116,117,118]. Similarly, findings by Ramin-Wright et al. in 2025 [119] demonstrate that sample entropy of oxygen saturation (SpO_2_-SampEn)—a nonlinear biomarker of physiological variability—is significantly lower in preterm infants and those with BPD, suggesting impaired autonomic regulation and increased vulnerability to hypoxemic events. These results support the potential utility of entropy-based metrics alongside HRV in monitoring corticosteroid effects and guiding individualized therapy.

**Figure 3:**
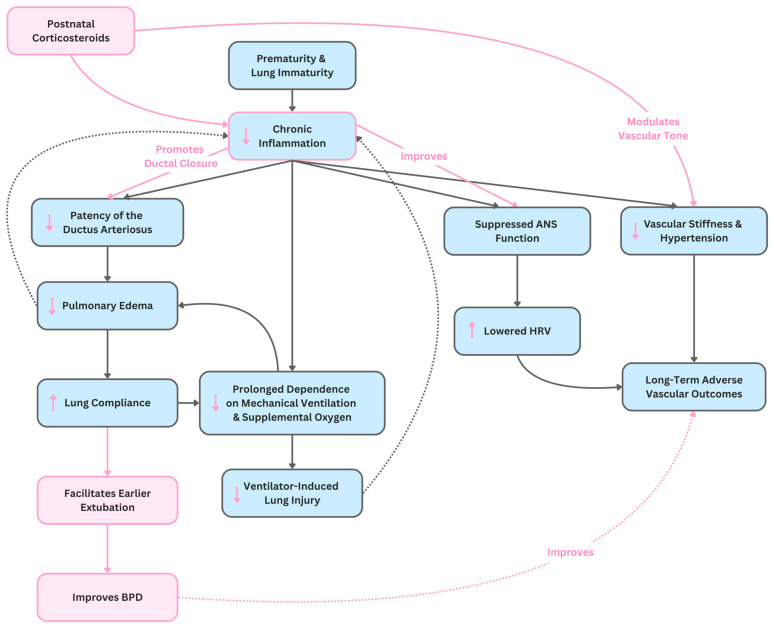
Integrated causal diagram of prematurity-related lung injury pathways and the modulatory effects of systemic corticosteroids on inflammation, ductus closure, vascular tone, and autonomic function. Pink arrows and boxes indicate proposed steroidal modulatory effects.

Thus, corticosteroids may help mitigate BPD-associated autonomic impairment, but further longitudinal studies are needed. HRV could emerge as a noninvasive biomarker for tracking steroid effects on neurodevelopment and cardiovascular health. Identifying patterns of autonomic dysfunction during steroid use may eventually help inform individualized therapy [120,121]. Figure 3 illustrates the proposed integrated pathways through which systemic corticosteroids modulate inflammation, vascular tone, ductus closure, and autonomic function in the context of prematurity-related lung disease. HRV has also been linked to extubation readiness, sepsis, and general physiological resilience in preterm infants [108,122]. It can be analyzed through time domain (e.g., SDNN, RMSSD), frequency domain (LF, HF, LF/HF ratio), and nonlinear metrics (e.g., Poincaré plots, entropy) [123,124,125].

Further investigation is warranted, as HRV may be a valuable tool to understand heart rate modulation, guide extubation decisions, and assess autonomic health during corticosteroid therapy in this vulnerable population. Despite these physiological insights, steroid-specific HRV evidence in preterm infants remains sparse and requires further validation.

The recognized short-term and long-term cardiovascular effects discussed throughout this section are summarized in Table 1.

## 3. Modalities of Interest for Monitoring Postnatal Corticosteroids Impacts on the Heart

While these advanced modalities offer high sensitivity, it is crucial to recognize that they are currently investigational research tools with no validated clinical decision thresholds for routine bedside use.

### 3.1. Two-Dimensional and Four-Dimensional Speckle Tracking Echocardiography (STE)

**Figure 4:**
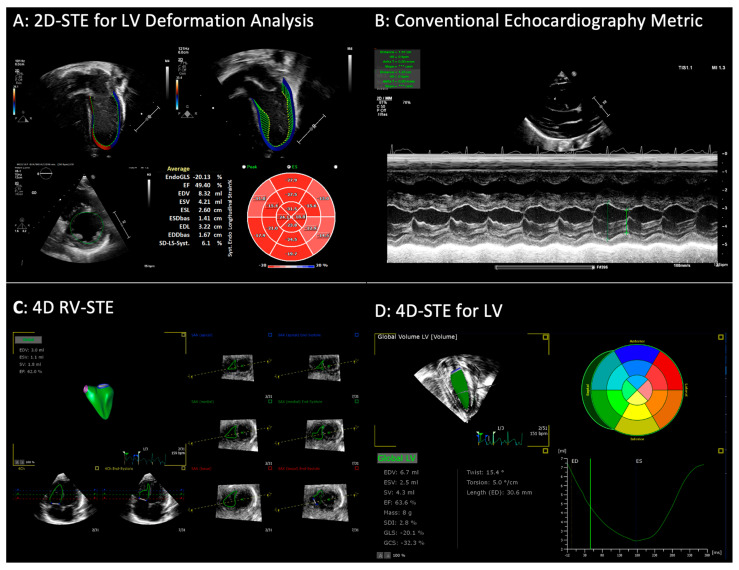
Sample echocardiographic images with 2D and 4D speckle tracking of LV and RV. Abbreviations: 2D (bi-dimensional); 4D (four-dimensional); LV (left ventricle); RV (right ventricle); MMode (motion-mode); STE (speckle tracking echocardiography).

Quantitative echocardiography techniques such as 2D and 4D Speckle Tracking Echocardiography (STE) enable a detailed assessment of ventricular dimensions, mass, and function to detect cardiac alterations or reduced heart function [17]. STE tracks myocardial motion over time thereby providing dimensional and functional insights into the RV, LV, and atria, while also enabling 4D reconstruction of volumes, leading to dynamic, time-resolved estimations of function and size. Strain analysis quantifies deformation, offering an early, sensitive marker of subclinical dysfunction, often detecting impairments before conventional functional metrics reveal abnormalities [18,19]. Several studies have explored the utility of these advanced echocardiographic tools in adult and neonatal populations. Levy et al. demonstrated how echocardiography, incorporating tissue Doppler and strain imaging, can effectively guide the assessment and management of neonatal heart failure unrelated to congenital heart disease [34]. Their findings emphasize the relevance of functional, not just structural, metrics in hemodynamic evaluation. Wu and Takeuchi reviewed real-world applications of 3D echocardiography [126], underscoring its role in dynamic chamber quantification, valvular assessment, and strain-derived insights across various populations, with clear potential for neonatal adaptation. Three-dimensional echocardiography allows the measurements and tracking of diastolic volumes to determine whether the size of the LV diminishes due to reactive concentric hypertrophy (ex. LV mass measurements using 3D echocardiography outlined in Figure 4); however, it is important to note that a concomitant reduction in pulmonary blood flow (ex. in closure of the PDA) would decrease preload to the LV and may also contribute to reductions in diastolic cavity size. Jahn et al. employed 2D STE in adults with end-stage renal disease [127], showing that global longitudinal strain (GLS) abnormalities could detect early myocardial dysfunction before changes in ejection fraction, and could independently predict cardiovascular mortality. Finally, in a large low-risk population, Biering-Sørensen et al. showed that impaired GLS, even in the absence of clinical disease, predicted long-term cardiovascular events [128], highlighting strain imaging as a sensitive and prognostically valuable modality. Leveraging not only conventional 2D echocardiography to assess PDA status but also advanced quantitative techniques such as STE is paramount to clarify the effects of systemic postnatal steroids on cardiac function and dimensions in preterm infants to better inform clinical decision-making and optimize therapeutic strategies. Future studies should prioritize longitudinal evaluation of cardiac behavior in infants exposed to steroids observing specifically response or non-response reflected in indicators such as ductal status, pulmonary phenotype and extubation success before, throughout and after steroidal exposure. One study is currently exploring this differential cardiac behavior—NORDIC-SPEC study: Surveillance of Postnatal Steroids Effects on Cardiac Function in Extremely Preterm Infants With Evolving Lung Disease (ClinicalTrials.gov ID NCT04644094).

### 3.2. Blood-Speckle Tracking Echocardiography (BSTE)

Another promising noninvasive imaging modality for neonatal cardiac assessment is blood speckle tracking echocardiography (BST), also referred to as blood speckle imaging. BST leverages high-frame-rate ultrasound to visualize and quantify intracardiac blood flow patterns in real time, offering detailed insights into cardiac hemodynamics and ventricular function [129]. In the neonatal and pediatric population, BST is emerging as a potential tool leveraging flow dynamics such as turbulent, laminar flow and vortices to assess intracardiac flow in congenital heart disease [130]. Preliminary studies have shown BST is sensitive in detecting pulmonary vortex formation (a surrogate for RV dysfunction) in children with sickle-cell disease [131]. Given its potential to detect early signs of altered right ventricular mechanics or pulmonary vascular loading—both relevant to BPD pathophysiology and potentially modifiable by postnatal corticosteroids, BST could provide novel insights in these neonates. However, the clinical utility of BST specifically in the context of BPD and steroids remains largely theoretical. Critically, normative BST data in neonatal preterm populations are sparse, and significant variability exists in acquisition protocols and interpretation [129]. Before BST can be reliably used to monitor disease progression or therapeutic response in BPD, comprehensive studies are needed to establish baseline cardiac flow patterns in healthy preterm neonates and to standardize imaging methodologies across centers. BST remains, for now, vendor-dependent, with only specific software and acquisition platforms allowing its use in the 2D plane. New strategies have also been developed to quantify vortex formation, vorticity, and flow energy loss (FEL) using vendor-independent Doppler velocity reconstruction (DoVeR) [132,133]. This approach utilizes conventional color Doppler to estimate these parameters without relying on specific machines or software brands. These avenues may also be explored to evaluate subtle changes in fluid mechanics following steroid exposure in preterm infants, providing insights into alterations—particularly in the context of reactive myocardial hypertrophy, changes in respiratory compliance, ventricular preload, and ductal physiology.

### 3.3. Tracking Saturation Profiles with NIRS

Another emerging noninvasive monitoring strategy for multi-organ saturation profiles is near-infrared spectroscopy (NIRS) [134]. NIRS machines are continuous regional monitors providing real-time tissue veinous-weighted oxygen saturations. NIRS is increasingly applied in preterm neonates to monitor regional tissue oxygenation, most extensively in the frontal cerebral cortex, where reference cerebral saturation values are well established and integrated into multiparametric brain monitoring [135,136,137,138,139]. Although NIRS use to assess lung oxygenation is much more recent, preliminary studies demonstrate that pulmonary NIRS reliably reflects changes in lung tissue oxygenation and correlates with arterial blood gases more accurately than pulse oximetry in preterm infants [140,141]. In neonatal respiratory disease, lung regional oxygen saturation measured by NIRS has shown promise for differentiating conditions such as transient tachypnea and pneumonia and correlating with disease severity and respiratory support requirements [142,143]. Importantly, while it is hypothesized that continuous pulmonary NIRS could detect evolving BPD and monitor treatment responses, including to postnatal corticosteroids, data specific to this application are lacking. NIRS has the potential to provide a more comprehensive view of oxygen saturation and intermittent hypoxia trends across multiple organs such as the lungs, brain, kidneys, and gut, offering a global perspective on the effects of postnatal steroids in neonates. Longitudinal studies and standardized protocols are needed to validate NIRS as a predictive and monitoring tool for BPD and to clarify how evolving lung disease and steroid therapy impact pulmonary oxygenation patterns in preterm infants.

### 3.4. Biofluid Markers

In addition to the conventional clinical and imaging modalities described above, several emerging biofluid markers have shown promise for monitoring infants’ responses to postnatal steroid therapy and guiding individualized treatment decisions. Infants with BPD have been shown to have elevated B-type natriuretic peptide (BNP) and N-terminal pro BNP (NTproBNP) levels. BNP is a hormone (NTproBNP is an inactive fragment of BNP) produced by the heart in response to stretching of the myocardium (i.e., as a response to increased blood volume and/or pressure) [144]. Additionally, NTproBNP levels are also known to be related to the severity of BPD and the development of pulmonary hypertension due to right ventricular strain [145,146]. As such, it is being explored as an early biomarker for BPD as groups have shown its levels increase with increased BPD severity. One group has shown that at 14 days of life NTproBNP is higher in those who later develop BPD, interestingly regardless of the presence of a significant PDA [147]. In addition, a study performed in 25 healthy, adult male volunteers observed the effects of a short course of dexamethasone on multiple cardiovascular biomarkers [148]. They found an increase in BNP levels in response to dexamethasone. Even though a variety of studies have attempted to ascertain normal ranges for BNP levels in preterm neonates [149,150,151], they have yet to be described in the preterm population receiving postnatal steroids in the context of evolving BPD. BNP is not alone: an additional set of cardiac biomarkers have also been documented to increase in situations of elevated cardiac stress, RDS and BPD such as atrial natriuretic peptide (ANP) and troponin [152,153,154,155] but their levels have yet to be tracked in response to postnatal steroid exposure. Given these observations, systematically tracking biomarker levels, starting with BNP and NT-proBNP, for the duration of longitudinal studies assessing the effects of postnatal corticosteroid therapy could be valuable in elucidating how steroid exposure influences cardiovascular stress or to help guide individualized treatment strategies.

Additionally, because BPD presents a multifactorial phenotype that both influences and is influenced by cardiac and pulmonary conditions as described above, various inflammatory and vasoactive biomarkers have been shown to increase in response to situations with systemic hypoperfusion and pulmonary over-circulation. For instance, in cases of a hemodynamically significant PDA where pulmonary overload or cerebral, splanchnic and renal ischemia may occur, elevated levels of vasoactive biomarkers such as endothelin-1 (CT-pro-ET-1), isoprostanes and copeptin have been documented [156]. Monitoring these biomarkers in response to postnatal steroid treatment could provide valuable insights into the effects of steroids on cardiac stress and BPD progression in extremely preterm infants.

Currently, with a variable definition of BPD itself, there is no standard biomarker-based diagnostic strategy for BPD. However, there are some research groups making strides to narrow the search to detect BPD earlier. One group has demonstrated the potential for 3 plasma protein markers (sialic acid-binding Ig-like lectin 14 (SIGLEC-14), basal cell adhesion molecule (BCAM), angiopoietin-like 3 protein (ANGPTL-3)) to enable early risk stratification of preterms with BPD using proteome screening and enzyme-linked immunosorbent assay (ELISA) in the first week of life [157]. Others have focused efforts in using umbilical cord blood (UCB), urine or tracheal aspirate (TA) samples [158,159]. Additionally explored markers include inflammatory markers, genomic and epigenetic markers, angiogenic growth factors, epithelial factors and more [160]. However, larger, multi-center clinical trials that incorporate multimodal assessments are needed before these approaches can be applied in clinical practice. Moreover, establishing baseline values for the most promising biomarkers could ultimately help identify meaningful deviations when infants are exposed to postnatal steroids; however, this remains an area requiring further research before it can be translated into practice.

### 3.5. MicroRNAs

Emerging evidence suggests that microRNAs (miRNAs), small non-coding RNAs regulating post-transcriptional gene expression, may play an important role in the pathogenesis and progression of BPD in preterm infants. Prior work has identified differential expression of several miRNAs in tracheal aspirates of preterm neonates with evolving BPD, which has been shown to promote lung injury and fibrosis [161]. In parallel, studies in adults with chronic obstructive pulmonary disease have demonstrated that corticosteroid treatment can significantly alter miRNA profiles. Exposure to the steroid fluticasone propionate has been linked to modulation of miRNAs involved in inflammatory and remodeling pathways, including miR-320d being identified as a novel mediator of inhaled corticosteroids, regulating the pro-inflammatory response of the airway epithelium [162]. However, data has yet to be published surrounding the role of microRNAs in the context of preterm neonates receiving steroids postnatally for evolving BPD. As such, it will be important to consider tracking miRNA signatures longitudinally in preterm infants receiving postnatal steroids to better characterize their dynamic responses and identify potential biomarkers or mechanistic targets relevant to both efficacy and adverse effects of treatment. Table 2 provides a summary of concomitant monitoring tools for postnatal corticosteroids effects on the cardiovascular system in preterm infants with evolving BPD.

## 4. Conclusions

Systemic postnatal corticosteroids remain a cornerstone in the management of evolving bronchopulmonary dysplasia in extremely preterm infants. Crucially, postnatal corticosteroids unequivocally influence ductal patency, frequently promoting the accelerated constriction and closure of the patent ductus arteriosus in the preterm population. However, their cardiovascular implications ranging from reactive hypertrophic cardiomyopathy to systemic hypertension, vascular stiffness, and autonomic dysregulation underscore the complexity of their physiological reach. Despite their widespread use, data characterizing individualized cardiovascular status before, during and after steroid exposure remains limited. Integrating longitudinal monitoring strategies into study protocols may help address uncertainties surrounding optimal dosing regimens, timing, and the lack of standardized physiological markers to guide care. Emerging technologies such as advanced echocardiographic modalities (3D and 4D imaging, strain analysis, blood speckle tracking), near-infrared spectroscopy, ECG-based heart rate variability analysis, biofluid biomarkers and microRNAs offer promising avenues for real-time, noninvasive evaluation of cardiac status, structure, function, and autonomic tone. These tools may enable more precise characterization of steroid-related cardiovascular effects and facilitate tailored therapeutic strategies. Integrating these modalities into both research and clinical workflows will be essential for transitioning from empiric to precision-based neonatal cardiovascular care. Ultimately, while integrating these advanced modalities represents a promising shift toward precision-based care, it is essential to recognize that the evidence for individualized steroid titration based on cardiovascular monitoring remains currently hypothetical and lacks validated clinical decision thresholds for routine bedside use.

## 5. Directions for Future Research

Despite growing recognition of the cardiovascular and autonomic effects of systemic postnatal corticosteroids in extremely preterm infants, significant knowledge gaps remain. To advance toward individualized corticosteroid therapy, future research must adopt a multisystem, longitudinal approach that captures the evolving interplay between cardiac structure, vascular function, and autonomic regulation across the course of treatment. Key priorities for future investigation include:

- Characterizing the temporal effects of corticosteroids on cardiac structure and function, including reactive hypertrophic remodeling, ductal dynamics, and vascular stiffness, using serial echocardiographic assessments (e.g., 3D/4D imaging, strain analysis, blood speckle tracking).
- Defining normative trajectories and thresholds for cardiovascular and autonomic markers such as heart rate variability and regional oxygen saturation in steroid-treated versus untreated infants.
- Correlating cardiovascular changes with systemic and pulmonary responses, to identify physiologic phenotypes of steroid responders and non-responders.
- Integrating multimodal data including imaging, electrophysiology, and biofluid biomarkers into predictive models that support real-time, individualized steroid decision-making.

This research trajectory will not only refine our understanding of steroid-related cardiovascular physiology but also complement pulmonary investigations by bridging heart-lung interactions. Part three of this series will examine how systemic corticosteroid exposure shapes growth and neurodevelopmental trajectories in extremely preterm infants—particularly through the lens of brain maturation and long-term developmental outcomes. By embracing an integrated, precision-based framework, future studies can help tailor corticosteroid therapy to the unique developmental trajectory of each infant supporting safer, more effective neonatal care.

## Figures and Tables

**Table 1:** Summary of short-term and long-term cardiovascular effects of postnatal corticosteroids in preterm infants.

Category	Short-Term/Acute Effects	Long-Term/Potential Effects
Myocardium	Transient reactive hypertrophy; thickening of the IVS and LVPW; potential for dynamic LVOT obstruction	Typically complete resolution of hypertrophy within 2–4 weeks post-discontinuation
Ductus Arteriosus	Accelerated ductal constriction and increased rates of spontaneous closure	Reduced need for surgical ligation or NSAID rescue therapies
Vascular System	Acute rise in systemic vascular resistance and blood pressure; treatment of refractory hypotension	Limited human evidence of long-term BP changes at school age; potential vascular stiffness (animal/adult models)
Autonomic System	Transient modulation of HRV and HRC indices	Uncertain; potential influence on neurodevelopmental trajectories and physiological resilience

**Table 2:** Summary table of concomitant monitoring tools for postnatal corticosteroids effects on the cardiovascular system in preterm infants with evolving BPD.

Monitoring Tool	Clinical Parameters Assessed	Strengths	Limitations
Conventional Echo (2D, M-Mode, color Doppler and tissue Doppler imaging (TDI))	Cardiac structure (chamber size, wall thickness, valve morphology), PDA status (patency, direction, shunt volume), ventricular systolic (EF, fractional shortening) and diastolic function (via TDI), pulmonary pressures (TR jet, septal flattening), pericardial effusion.	- Widely available and validated - Bedside evaluation of ductal and cardiac function - Doppler and TDI enhance flow and diastolic assessment - Familiar and fast in more NICUs	- Limited reproducibility of EF and M-mode in preterms - Misses early/subclinical dysfunction - Operator-dependent - Cannot quantify regional strain or subtle injury
Speckle Tracking Echocardiography (STE)	Myocardial deformation (“strain”) in all chambers (LV, RV, atria), global longitudinal strain (GLS), ventricular twist, subclinical dysfunction, dynamic response to interventions (e.g., steroids, PDA closure), potential long-term prognostic utility.	- Highly sensitive to early injury - Quantitative and angle/load-independent - Detects steroid effects before EF declines - Regional strain tracking (e.g., basal/apical)	- Requires high image quality and post-processing - No normative data in extreme preterms - Time-consuming - Specialized expertise needed
3D Echocardiography	Volumetric chamber size and function (without geometric assumptions), EF, ventricular mass, dynamic valve and flow assessment (e.g., TR, MR), potentially useful for monitoring structural remodeling post-steroids.	- Superior accuracy for volumes and EF - Dynamic assessment of complex anatomy - 3D valve reconstruction possible - Valuable for precise serial measurements in research	- Limited NICU availability - High operator expertise needed - No preterm reference data - Motion artifacts in small/tachycardic infants
Blood-Speckle Tracking Echocardiography (BSTE)	Intracardiac flow patterns (laminar, turbulent, vortex), potential surrogate for RV dysfunction or altered pulmonary loading relevant to BPD pathophysiology and steroid effects.	- Visualizes real-time flow dynamics - Sensitive to early RV changes (e.g., pulmonary vortex) - Potential for novel insights in steroid response	- Still experimental in neonates - Sparse normative data and high inter-site variability - Requires standardization before clinical use
Electrocardiogram (ECG) and Heart Rate Variability (HRV)	Autonomic nervous system (ANS) activity, stress response, extubation readiness, sepsis risk, systemic inflammation.	- Noninvasive, continuous - Reflects ANS tone and steroid impact	- Affected by many confounders (infection, meds) - No steroid-specific protocols - Interpretation complex in unstable infants
Near- Infrared Spectroscopy (NIRS)	Regional oxygenation of brain, lung, and other tissues; early marker of evolving BPD or hemodynamic shifts with steroid use.	- Continuous, real-time - Sensitive to subtle changes - Established in brain monitoring, expanding to lung	- Limited validation for pulmonary use - Motion/prone to artifact - No defined intervention thresholds
Biofluid Markers	BNP, NT-proBNP, troponin, ANP, etc.; reflect cardiac strain, BPD severity, PH risk, systemic stress response.	- Objective biochemical signals - Early risk stratification potential - BNP may reflect steroid-induced stress	- Normative values lacking in preterms on steroids - Influenced by PDA, PH, RDS - Not validated for therapy guidance
Micro- RNAs	Gene regulation markers of inflammation, injury, and steroid response.	- Insight into molecular mechanisms - May enable personalized steroid dosing - Potential for treatment monitoring	- No clinical neonatal data - Requires advanced lab capabilities - Investigational only

Abbreviations: 2D (two-dimensional); 3D (three-dimensional); ANP (atrial natriuretic peptide); BPD (bronchopulmonary dysplasia); BNP (B-type natriuretic peptide); BSTE (blood speckle tracking echocardiography); ECG (electrocardiogram); EF (ejection fraction); GLS (global longitudinal strain); HRV (heart rate variability); LV (left ventricle); MR (mitral regurgitation); M-Mode (motion mode); NICU (neonatal intensive care unit); NIRS (near-infrared spectroscopy); NT-proBNP (N-terminal pro-B-type natriuretic peptide); PDA (patent ductus arteriosus); PH (pulmonary hypertension); RDS (respiratory distress syndrome); RV (right ventricle); STE (speckle tracking echocardiography);; TDI (tissue doppler imaging); TR (tricuspid regurgitation).

## Data Availability

No new data were created.

## Abbreviations

The following abbreviations are used in this manuscript:

2D	Two-dimensional
4D	Four-dimensional
ALPM	anterolateral papillary muscle
ANP	atrial natriuretic peptide
Ap4C	apical four-chamber view
BPD	bronchopulmonary dysplasia
BNP	B-type natriuretic peptide
BSTE	blood speckle tracking echocardiography
CT-pro-ET-1	C-terminal pro-endothelin-1
Dexa	dexamethasone
EF	ejection fraction
GA	gestational age
GC	glucocorticoid
HC	hydrocortisone
HRV	heart rate variability
IVS	interventricular septum
LVPW	left ventricular posterior wall
LV	left ventricle
MC	mineralocorticoid
mPSAX	modified parasternal short-axis view
MV	mechanical ventilation
M-Mode	motion-mode
NIRS	near-infrared spectroscopy
NSAIDs	non-steroidal anti-inflammatory drugs
NT-proBNP	N-terminal pro-B-type natriuretic peptide
PDA	patent ductus arteriosus
PLAX	parasternal long-axis view
PMA	post-menstrual age
PMPM	posteromedial papillary muscle
RDS	respiratory distress syndrome
RV	right ventricle
STE	speckle tracking echocardiography
TDI	tissue Doppler imaging
TR	tricuspid regurgitation
UCB	umbilical cord blood
